# Viral Bimolecular Fluorescence Complementation: A Novel Tool to Study Intracellular Vesicular Trafficking Pathways

**DOI:** 10.1371/journal.pone.0125619

**Published:** 2015-04-27

**Authors:** Brennan S. Dirk, Rajesh Abraham Jacob, Aaron L. Johnson, Emily N. Pawlak, P. Craig Cavanagh, Logan Van Nynatten, S. M. Mansour Haeryfar, Jimmy D. Dikeakos

**Affiliations:** Department of Microbiology and Immunology, The University of Western Ontario, Schulich School of Medicine and Dentistry, London, Ontario, Canada; Helmholtz Zentrum Muenchen - German Research Center for Environmental Health, GERMANY

## Abstract

The Human Immunodeficiency Virus type 1 (HIV-1) accessory protein Nef interacts with a multitude of cellular proteins, manipulating the host membrane trafficking machinery to evade immune surveillance. Nef interactions have been analyzed using various *in vitro* assays, co-immunoprecipitation studies, and more recently mass spectrometry. However, these methods do not evaluate Nef interactions in the context of viral infection nor do they define the sub-cellular location of these interactions. In this report, we describe a novel bimolecular fluorescence complementation (BiFC) lentiviral expression tool, termed viral BiFC, to study Nef interactions with host cellular proteins in the context of viral infection. Using the F2A cleavage site from the foot and mouth disease virus we generated a viral BiFC expression vector capable of concurrent expression of Nef and host cellular proteins; PACS-1, MHC-I and SNX18. Our studies confirmed the interaction between Nef and PACS-1, a host membrane trafficking protein involved in Nef-mediated immune evasion, and demonstrated co-localization of this complex with LAMP-1 positive endolysosomal vesicles. Furthermore, we utilized viral BiFC to localize the Nef/MHC-I interaction to an AP-1 positive endosomal compartment. Finally, viral BiFC was observed between Nef and the membrane trafficking regulator SNX18. This novel demonstration of an association between Nef and SNX18 was localized to AP-1 positive vesicles. In summary, viral BiFC is a unique tool designed to analyze the interaction between Nef and host cellular proteins by mapping the sub-cellular locations of their interactions during viral infection.

## Introduction

The sub-cellular localization of mammalian proteins is coordinated by the membrane trafficking machinery, including a vast network of membrane-bound vesicles and adaptor molecules [1, 2]. Viruses, such as Human Immunodeficiency Virus type 1 (HIV-1), are able to exploit the host membrane trafficking machinery and key cellular components to favour viral replication. HIV-1 produces 15 viral proteins [3, 4], including a 27 kDa accessory protein termed Nef, which lacks any known enzymatic activity, but is essential for viral pathogenesis [5, 6]. Nef mediates its pathogenic effects by modulating membrane trafficking in infected cells. Notably, Nef facilitates downregulation of various cell surface molecules, including major histocompatibility complex-I (MHC-I), which results in attenuation of the immune response by impairing the presentation of viral antigens to cytotoxic T-lymphocytes (CTLs) [7, 8].

Nef-mediated MHC-I downregulation is primarily orchestrated by protein-protein interactions between Nef and various host cellular proteins [7, 9–12]. This includes the membrane trafficking regulators phosphofurin acidic cluster sorting proteins 1 and 2 (PACS-1 and PACS-2), which form specific protein complexes with Nef at distinct sub-cellular locations in order to downregulate MHC-I [7, 9, 12, 13]. In turn, PACS-1 can specifically interact with the coat adaptor protein-1 (AP-1) to facilitate Nef mediated sequestration of MHC-I away from the cell surface [7, 13, 14]. The PACS-1/AP-1 interaction, as well as the crystal structure of Nef in complex with AP-1 and MHC-I, demonstrate that host membrane trafficking regulator proteins, such as AP-1 and PACS-1, are key for HIV-1 immune evasion [14]. Recently, the interactions between Nef and PACS proteins have been visualized using bimolecular fluorescence complementation (BiFC). BiFC is a microscopy technique that localizes protein interactions through the reconstitution of a functional fluorophore upon the interaction of two protein-binding partners each fused to a non-fluorescent fragment of a fluorophore [15–17]. Although BiFC has demonstrated that PACS-1 or PACS-2 and Nef interact at distinct sub-cellular compartments, it has not been shown with concurrent expression from a single plasmid or in the context of viral infection with other HIV-1 proteins present [9].

This study addresses the current limitations of using BiFC to investigate viral protein interactions through the development of a lentiviral vector that enables simultaneous expression of Nef with various binding partners from the same vector in the context of a viral infection. To accomplish this, we utilized a lentiviral expression system yielding pseudovirions modified such that they only undergo a single round of replication, but are still capable of genomic integration [18]. The co-expression of transgenes of interest was achieved by inserting the autocleavable 2a (F2A) coding sequence from the foot and mouth disease virus into the previously described HIV-1 based vector pNL4-3 Δgag/pol eGFP [19–22]. Previous reports have demonstrated that insertion of an F2A site stalls translation, resulting in the production of cleaved polyproteins containing a 21 residue carboxy terminal F2A tag and a single proline addition at the amino terminus [23]. We have used this unique system to express multiple transgenes fused to split fluorophores, thereby permitting analysis of protein-protein interactions using BiFC. Our results demonstrate that viral BiFC can be used to study the interaction between HIV-1 Nef and PACS-1 at both early and late endosomal compartments. The utility of viral BiFC is highlighted by its ability to provide the distinct sub-cellular localization of the interaction between Nef and MHC-I. In addition, viral BiFC can be used to study novel interactions between Nef and host membrane trafficking regulators. Indeed, using viral BiFC we demonstrate for the first time an interaction between Nef and the sorting nexin 18 (SNX18) protein. Viral BiFC represents a unique tool enabling the visualization of Nef interactions at specific sub-cellular locations in the context of an HIV-1 infection.

## Materials and Methods

### Cell Culture

HeLa (ATCC, Manassas, VA) and HEK 293T cells (Life Technologies, Carlsbad, CA) were grown in complete DMEM containing 10% fetal bovine serum (Life Technologies, Waltham, WA), 100μg/ml penicillin-streptomycin (Hyclone, Logan, UT), 1% sodium pyruvate, 1% non-essential amino acids and 2mM L-glutamine (Hyclone). Jurkat E6.1 T-cells (Catalog number 177; National Institutes of Health, AIDS Research and Reference Reagent Program) were cultured in RPMI 1640 with supplements as mentioned above. All cell lines were grown at 37°C in the presence of 5% CO_2_ and sub-cultured in accordance with supplier’s recommendations.

### Proviral plasmids and cloning strategy

**Fig 1:**
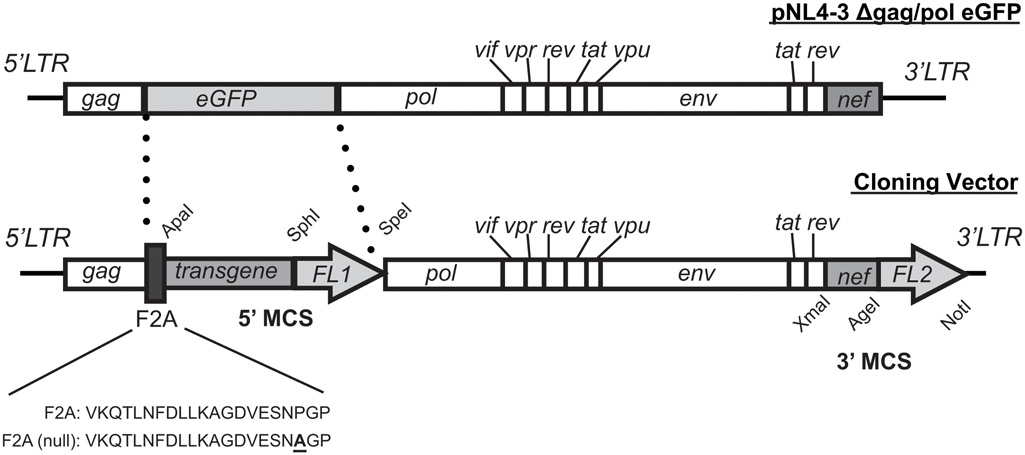
Construction of an HIV-1 derived lentiviral expression system harboring an F2A peptide and two multiple cloning sites. The pNL4-3 Δgag/pol eGFP vector (top) was engineered to contain the self-cleaving F2A peptide followed by a 5’ MCS (ApaI, SphI and SpeI), to introduce various transgene fusion proteins of interest. A MCS was introduced at the 3’ end in order to insert various Nef fusion proteins (XmaI, AgeI and NotI). (MCS: multiple cloning site; *FL1*: fluorophore fused to transgene of interest in the 5’ MCS; *FL2*: fluorophore fused to Nef in the 3’ MCS).

3’ cloning site for Nef fusion proteins: The previously described pNL4-3 Δgag/pol eGFP replication incompetent HIV-1 proviral vector [19, 20] was used as the base template for modification into our final expression vector system. First, primer overlap extension mutagenesis [24] was used to amplify two fragments flanking the Nef coding sequence in order to remove Nef and insert XmaI, AgeI and NotI restriction sites, termed the 3’ multiple cloning site (MCS). Specifically, an initial PCR reaction (Reaction I) was performed with primers JD 14 and JD 37 (Table 1) in order to amplify a 316 bp fragment upstream of Nef, containing the 3’ MCS restriction sites. A subsequent PCR reaction (Reaction II) amplified a 1157 bp fragment immediately after the Nef stop codon using primers JD 38 and JD 15 (Table 1). The forward primer in Reaction II contained complementary nucleotides to the 3’ MCS restriction sites in JD 37. Products from Reaction I and II were purified, mixed and amplified (Reaction III) using the flanking JD 14 and JD 15 primers (Table 1). The Reaction III product was inserted into the pNL4-3 Δgag/pol eGFP base vector using BamHI and NcoI restriction sites in order to generate a pNL4-3 Δgag/pol eGFP with the 3’ MCS in lieu of Nef (Fig 1). This 3’ MCS is capable of accepting Nef fusion proteins with various fluorophores. The mStrawberry (mSB) fragment was amplified from Addgene plasmid 20970 [25]. For the BiFC experiments regions expressing the amino portion of the Venus fluorophore (V_N_; amino acids 1–173) or the carboxy portion of the Venus fluorophore (V_C_; amino acids 155–238) [15] were inserted in either MCS.

**Table 1:** Primers used to construct the lentiviral expression vector.

Primer	Sequence
JD 14	GTGAACGGATCCTTAGCAC
JD 15	CCTGCACTCCATGGATCA
JD 37	CCTTGGGCGGCCGCATATACCGGTAAATTTCCCGGGCTTATAGCAAAATCCTTTCCAAGCCCT
JD 38	CCTTGGCCCGGGAAATTTACCGGTATATGCGGCCGCCATCGAGCTTGCTACAAGGGAC
JD 42	GTTGTTGCAGAATTCTTATTATGGCTTCCAC
JD 43	CCTTGGTTGGCCAGGGCCCGTAAAAACAGTACATACAGACAATGGC
JD 106	TAGTGAAACAGACTTTGAATTTTGACCTTCTCAAGTTGGCGGGAGACGTGGAGTCCAACCCCGGGCCCGCAGCAGCATGCGCAGCAACTAGTTAATGGCC
JD 107	CTTAACTAGTTGCTGCGCATGCTGCTGCGGGCCCGGGGTTGGACTCCACGTCTCCCGCCAACTTGAGAAGGTCAAAATTCAAAGTCTGTTTCACTACATG
JD 132	GGTTGGGCATGCATGGCGCTGCGCGCCCGG
JD 142	GCAGCAACTAGTTTACTTATCGTCGTCATCCTTGTAATCTCCGTCCTCGATGTTGTGGCGGATC
JD 144	GCTGCTGCATGCATGGCCGTZATGGCGCCC
JD 165	TGAAGCGCGCACGGCAAG
JD 166	TGCTGCGGGCCCGGCGTTGGACTCCACGTCTCCCGC

### Generation of the F2A cleavage site and 5’ MCS

An ApaI site was inserted directly after eGFP in the pNL4-3 Δgag/pol eGFP plasmid (JD 43 and JD 42). Briefly, a PCR reaction was conducted to produce the desired ApaI restriction site directly adjacent to the MscI restriction site already present in the proviral vector. The product was then digested with MscI and EcoRI and cloned into the parent vector. Subsequently, to insert the F2A sequences and the 5’ MCS, two complementary primers (JD 106 and 107, Table 1) were engineered to contain the F2A sequence (VKQTLNFDLLKAGDVESNPGP) in addition to ApaI, SphI and SpeI restriction sites, termed the 5’ MCS, plus a 2 bp overhang that is complementary to the ApaI and SphI cut sites. These primers were annealed together and ligated into pNL4.3 ΔGag/pol eGFP with the inserted ApaI cut site (Fig 1; bottom), which was cut with SphI and ApaI. The resulting plasmid contained unique 5’ restriction cut sites in order to permit insertion of foreign genes. To generate the F2A (null) control vector, primer overlap extension mutagenesis was performed to mutate the penultimate proline to an alanine. First, a forward primer was generated upstream of the F2A site containing a PauI restriction site (JD 165). Second, a reverse primer was engineered to contain the F2A mutation with an adjacent ApaI site (JD 166). A subsequent PCR was conducted using F2A mSB-Flag Nef-eGFP as a template, and the product was inserted into the F2A vector utilizing the PauI and ApaI restriction sites, thereby replacing the functionally active F2A with the non-functional mutant.

To generate the MHC-I (HLA-A2 allele) and SNX18 BiFC constructs, products were amplified from expression plasmids containing cDNA sequences for MHC-I-V_N_ (JD 144 and JD 142; Table 1) or SNX18-V_N_, (JD 132 and JD 142; Table 1, SNX18 cDNA was provided by Rytis Prekaris; University of Colorado Denver) then cloned into the viral vector using the SpeI and SphI cut sites. To engineer the F2A-MHC-I-mCherry ΔNef viral vector, we digested the parental backbone of F2A-MHC-I-mCherry Nef-GFP with PauI and EcoRI and cloned the product into a pNL4-3 ΔNef construct generating an F2A-MHC-I-mCherry ΔNef viral vector.

### Pseudovirus production and processing

Pseudovirions were produced in HEK 293T cells. Cells were triple transfected using PolyJet (FroggaBio, Toronto, ON) with pNL4-3 Δgag/pol eGFP or the modified variants, as well as pdR8.2 and pMD2.G as previously described [26]. Pseudovirus was harvested 48 hours post-transfection. Briefly, virus-containing media was first centrifuged at 3000xg for 5 minutes and subsequently filtered. The filtered supernatant was supplemented with an additional 10% FBS prior to storage at -80°C.

### Western Blots, Antibodies and Infections

Jurkat E6.1 T-cells or HeLa cells were infected with various pseudoviruses for 48 hours, at which point infected cells were washed once with phosphate buffered saline (PBS) and subsequently lysed in lysis buffer (0.5M HEPES, 1.25M NaCl, 1M MgCl_2_, 0.25M EDTA, 0.1% Triton X-100 and 1X complete Protease inhibitor Tablets (Roche, Indianapolis, IN). Cells were incubated on a rotator for 20 minutes at 4°C before removing insoluble cellular debris by centrifugation at 20,000xg for 20 minutes. Lysates were boiled at 98°C in 5X SDS-PAGE sample buffer (0.312M Tris pH 6.8, 25% 2-Mercaptoethanol, 50% glycerol, 10% SDS) and proteins were separated on a 12% SDS-PAGE gel and subsequently transferred to nitrocellulose membranes. Membranes were blocked in 5% non-fat skimmed milk (Bioshop, Burlington, ON) in TBST containing 0.1% Triton X-100 for 1 hour, then incubated overnight at 4°C with various antibodies: rabbit anti-Nef polyclonal antibody (1:4000; catalog number 2949, NIH AIDS Research and Reference Reagent Program, USA), rabbit anti-GFP polyclonal antibody (1:2000; Clontech; Mountain View, CA), rat anti-DYKDDDK monoclonal IgG (1:2500; BioLegend, San Diego, CA), or rabbit anti-mCherry monoclonal IgG (1:2000; Thermo Scientific). Membranes were then washed and incubated for two hours with the appropriate species-specific HRP-conjugated antibodies (1:5000; Thermo Scientific). All blots were developed and quantified using ECL substrates (Millipore Inc., Billerica, MA) and a C-DiGit chemiluminescence Western blot scanner (LI-COR Biosciences, Lincoln, NE).

Cleavage efficiency of the F2A site was calculated by dividing the signal intensity of the cleaved product by the sum of both the cleaved and un-cleaved product. Efficiencies were then normalized to the wildtype F2A cleavage. Subsequently, a ratio was obtained by comparing the wildtype functional F2A cleavage efficiency to the mutant.

For flow cytometry the following antibodies were used: W6/32 (anti-MHC-I; pan-selective, provided by D. Johnson, Oregon Health and Sciences University), antibody conjugated to APC/Cy7 (1:25, Biolegend, San Diego, CA), anti-p24 clone KC57 conjugated to RD1 (phycoerythrin) (1:50, Beckman Coulter, Brea, CA), anti BB7.2 conjugated to APC/Cy7 (1:25, Biolegend, San Diego, CA).

For immunofluorescence, rabbit anti-Rab5 (clone C8B1; 1:200, Cell Signaling), mouse anti-LAMP-1 (clone H4A3, 1:100, obtained from the Developmental Studies Hybridoma Bank) and mouse anti-AP-1γ (Sigma Aldrich) antibodies were used.

### Microscopy

HeLa cells were seeded onto sterile glass coverslips at 5x10^5^ cells per coverslip for 16 hours prior to infection. Cells were infected for 48 hours before processing for immunofluorescence. Briefly, cells were washed three times with PBS before fixation in 4% paraformaldehyde for 20 minutes at room temperature. Cells were subsequently washed with PBS twice prior to nuclear staining. Immediately prior to imaging, Hoechst nuclear stain (1μg/ml; Thermo Scientific) was added to the coverslip and incubated for 10 minutes. Cells were imaged on the Leica DMI6000 B on 63X objective using the FITC, CY3 and DAPI filter settings using the Hamamatsu Orca-flash 4.0 Camera.

For BiFC experiments, infections were set as above. Prior to fixation, the cells were incubated at room temperature for 2 hours to allow the reconstituted fluorophore to mature. The fixation protocol was then carried out as described above. To visualize early or late endosomes, cells were stained with rabbit anti-Rab5 or mouse anti-LAMP-1 antibodies, respectively. Samples were incubated in a permeabilization buffer containing 1% BSA in PBS and 0.2% Triton X-100 for 5 minutes, then blocked in a buffer containing 5% BSA in PBS for 1 hour. Anti-Rab5 or LAMP-1 antibodies were then diluted in 1% BSA and 0.2% Triton X-100 (1:200 and 1:100, Jackson ImmunoResearch). Cells were then washed three times in PBS (2 minutes each) before adding the secondary donkey anti-rabbit AlexaFluor 647 or donkey anti-mouse Alexafluor 647 (1:1000; Jackson ImmunoResearch) in the same manner as the primary antibody. AP-1 staining was carried out as mentioned above using mouse anti-AP-1γ (1:200; Sigma Aldrich) primary antibody and donkey anti-mouse Alexafluor 647 (1:1000; Jackson ImmunoResearch). Samples were washed three times in PBS (1 minute each) prior to imaging and mounted onto glass slides using DAPI Fluormount-G (Southern Biotech, Birmingham, AL). Cells were imaged on the Leica DMI6000 B on 100X objective using the FITC, CY5 and DAPI filter settings using the Hamamatsu Photometrics Delta Evolve camera. Images were subsequently deconvolved using the Advanced Fluorescence Deconvolution application on the Leica Application Suite software. Co-localization analysis was conducted using the Manders Coefficient and Pearson Correlation from the Image J plugin as described previously [27].

### Flow Cytometry

To quantify the cell surface expression levels of MHC-I, Jurkat E6.1 T-cells were infected with the appropriate viruses and 72 hours post infection cells were surface stained for MHC-I using W6/32 antibody conjugated to APC/Cy7 (Biolegend, San Digego, CA). Following fixation in 1% paraformaldehyde, cells were permeabilized with cold methanol. Subsequently, intracellular staining with RD1 (phycoerythrin) conjugated anti-p24 (Beckman Coulter) was performed to gate for infected cells. Cell surface MHC-I expression was quantified by flow cytometry (BD FACS Canto II) and the data analyzed using FlowJo software (version 9.6.4, Treestar, Ashland, OR).

The ability of the MHC-I-mCherry fusion protein to be trafficked to the membrane was tested using an allele specific antibody. Jurkat E6.1 T-cells were infected with the appropriate virus and 48 hours post-infection, cell surface staining was performed using the BB7.2 antibody (Biolegend) which recognizes only MHC-I molecules encoded by A*02 alleles. Cells were then fixed in 1% paraformaldehyde and permeabilized with cold methanol and intracellularly stained with RD1 (phycoerythrin) conjugated anti-p24 to gate for infected cells. MHC-I-mCherry cell surface expression was quantified by flow cytometry (BD FACS Canto II) and the data analyzed using FlowJo software (version 9.6.4)

### Statistics

All statistics were conducted using a paired T-test on Graph Pad Prism (Graph Pad Sofware Inc., La Jolla, CA).

## Results

### Designing a lentiviral vector enabling dual transgene expression

Multiple Nef-interacting proteins have been identified using *in vitro* interaction assays, cellular co-immunoprecipitation analyses, and more recently by fluorescence resonance energy transfer and mass spectrometry [7, 9, 10, 13, 28–35]. However, the Nef protein-protein interaction network has never been defined in the context of expression from a single vector that mimics the conditions present during a viral infection. To address this, we constructed a lentiviral vector containing an F2A cleavage site or the non-functional mutant, F2A (null), as a control, thereby facilitating concurrent expression of Nef and a potential Nef-interacting partner (Fig 1). Vector assembly was initiated using the previously described pNL4-3 Δgag/pol eGFP vector as a base (Fig 1; top panel) [19, 20]. This base vector contains intact 5’ and 3’ long terminal repeat (LTR) regions and expresses all HIV-1 viral proteins except full-length Gag and Pol. These genes are mutated in the base vector to generate truncated proteins and must be supplied in trans for productive pseudovirion synthesis (Fig 1; top panel) [20, 26]. To facilitate gene insertion, two multiple cloning sites (MCSs) were introduced into the base vector: a 3’ MCS containing the XmaI, AgeI and NotI restriction sites and a 5’ MCS containing the ApaI, SphI and SpeI restriction sites (Fig 1; bottom panel). We inserted HIV-1 Nef fused with the eGFP fluorescent tag into the 3’ MCS whereas the 5’ MCS was used to insert potential Nef-interacting partners.

### Insertion of an F2A site into a lentiviral vector allows concurrent protein production

**Fig 2:**
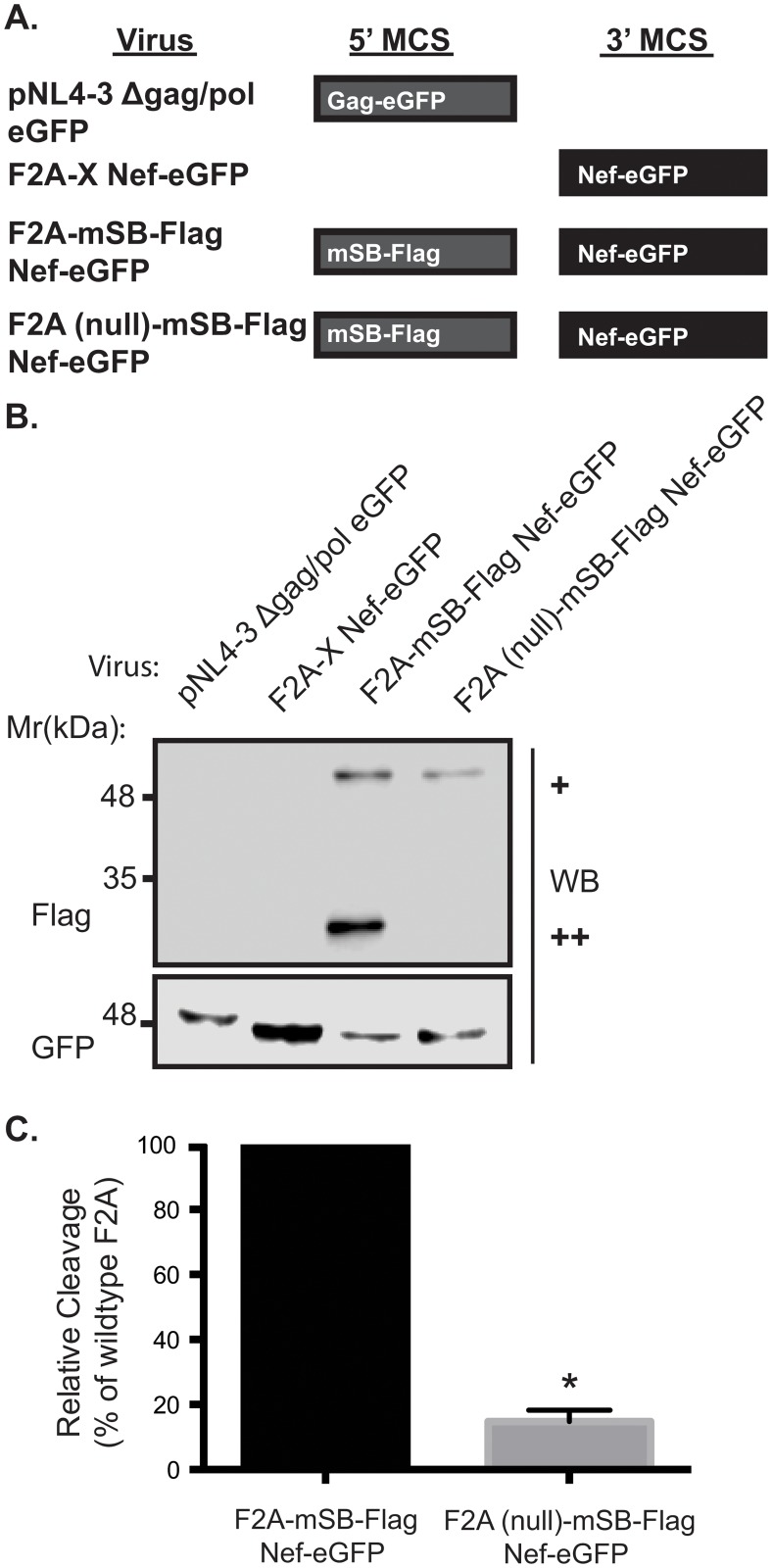
Functional cleavage at the engineered F2A site. Viruses were engineered with various proteins within the 5’ MCS and/or the 3’ MCS and Jurkat E6.1 T-cells were infected with the resulting pseudoviruses. Flag and GFP specific western blots were performed on lysates collected 48 hours post infection to verify protein expression levels. **(A)** Schematic representation of proteins produced from lentiviral expression system. **(B)** A Flag specific western blot was used to quantitate the cleavage efficiency at the F2A site in the F2A-mSB-Flag Nef-eGFP virus, compared to the F2A mutant, F2A (null)-mSB-Flag Nef-eGFP, which lacks cleavage activity (+ uncleaved product, ++ cleaved product). GFP specific western blots confirmed the presence of the Gag-eGFP fusion protein (lane 1) or Nef-eGFP fusion proteins (lanes 2–4). **(C)** Cleavage efficiency at the F2A site was 6-fold higher compared to the F2A (null) virus (* indicates *p*-value < 0.05). Details on how the cleavage efficiency was calculated are included in Materials and Methods. Error bars calculated from 3 independent experiments. *p*-value was determined by paired t-test.

To avoid the production of proteins fused to truncated Gag/Pol proteins from the 5’ MCS, we exploited the self-cleaving property of the 2A peptide (F2A) derived from the foot and mouth disease virus by inserting the 21 amino acid F2A site between the Gag/Pol fusion protein and the 5’ MCS (Fig 1; bottom panel). The resulting vector, pNL4-3 F2A-X Nef-eGFP (Fig 2A) has an empty 5’ MCS in order to accommodate future gene insertions. To test the cleavage efficiency of the F2A site, we constructed a lentiviral vector containing a Flag-tagged mStrawberry (mSB) fluorophore in the 5’ MCS (Fig 2A). Pseudovirus from the resulting pNL4-3 F2A-mSB-Flag Nef-eGFP construct was then used to infect Jurkat E6.1 T-cells. In order to directly quantitate the cleavage efficiency, a control vector with an inactive F2A site was constructed (Figs 1 and 2; F2A (null)). Site-directed mutagenesis of the penultimate proline residue to alanine has been demonstrated to render the F2A site inactive [36]. In agreement with cleavage mediated at the F2A site, western blot analysis demonstrated the production of a cleaved Flag-tagged mStrawberry protein that migrated just below the 35 kDa molecular weight marker (Fig 2B; ++ Flag blot). Consistent with mutation of the penultimate proline in the F2A site to produce a defective cleavage site [22, 36, 37], cells infected with pNL4-3 F2A (null)-mSB-Flag Nef-eGFP pseudovirus produced a Flag-tagged mStrawberry protein fused to the HIV-1 Gag/Pol protein that migrated just above the 48 kDa molecular weight marker (Fig 2B; + Flag blot). Quantification of the cleavage efficiency between the F2A and the F2A (null) site was determined by dividing the densitometry measurement of the cleaved product by the sum of both the cleaved and uncleaved products. We observed a 6-fold increase in cleavage efficiency in the presence of a functional F2A site, confirming that the cleavage is F2A-dependent (Fig 2C). Moreover, the pNL4-3 F2A-mSB-Flag Nef-eGFP construct efficiently produced Nef fused to the eGFP fluorophore, confirming that the 3’ MCS can also produce a fluorophore-tagged protein of interest (Fig 2B; lane 3 GFP blot). Overall, these results demonstrate the 3’ and 5’ MCSs can be used to produce conjugated proteins and the proteins expressed from the 5’ MCS are efficiently cleaved from truncated Gag/Pol.

### Nef and Nef-interacting partners are simultaneously expressed in a lentiviral vector

**Fig 3:**
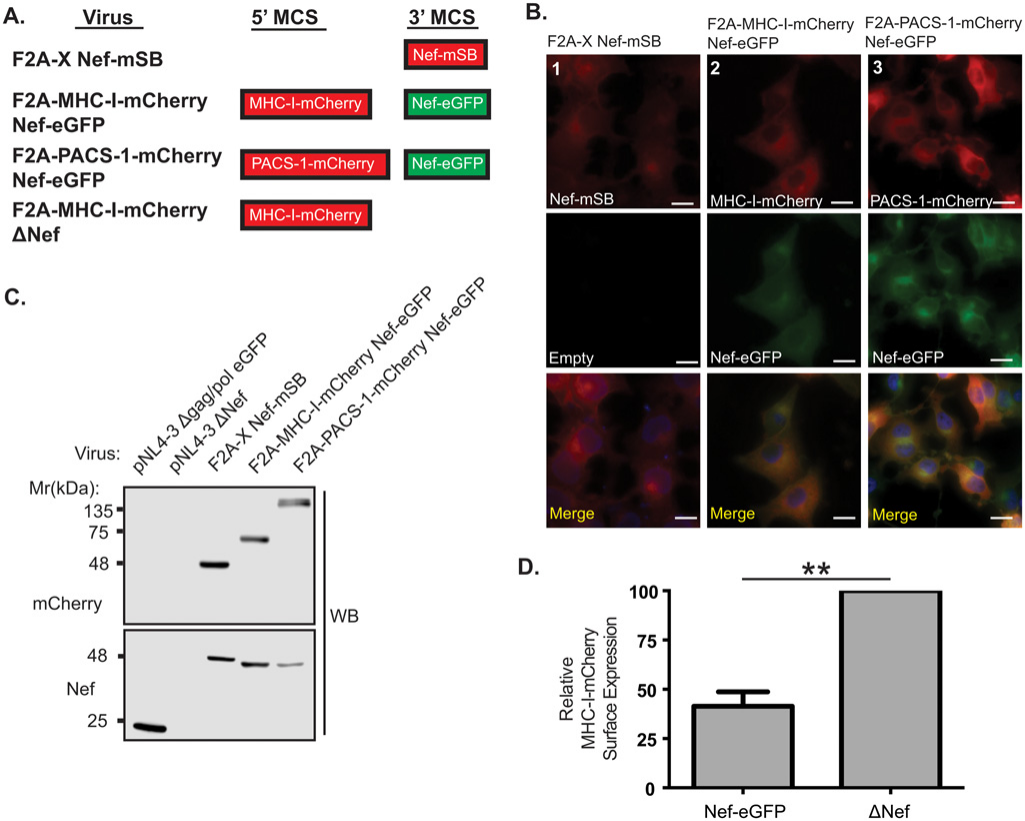
Fluorescence microscopy confirms expression of proteins from the 5’ and 3’ MCS. Viruses were engineered to produce MHC-I-mCherry or PACS-1-mCherry from the 5’ MCS in combination with Nef-eGFP from the 3’ MCS. **(A)** Schematic representation of proteins produced from lentiviral expression system. **(B)** To detect the fluorescent fusion proteins, HeLa cells were infected and visualized 48 hours post infection by widefield fluorescence microscopy. Expression of the Nef-mSB fusion protein was confirmed (column 1), along with concurrent expression of MHC-I or PACS-1-mCherry fusions with Nef-eGFP (columns 2 and 3). Cell nuclei were stained using Hoescht nuclear stain (blue). Scale bars represent 15μm. **(C)** mCherry and Nef specific western blots were performed to confirm the expression of the fusion proteins. **(D)** Jurkat E6.1 T-cells were infected with pNL4-3 F2A-MHC-I-mCherry Nef-eGFP (Nef-eGFP) or pNL4-3 F2A-MHC-I-mCherry ΔNef (ΔNef). At 48 hours post infection, cells were surface stained for MHC-I-mCherry (BB7.2 antibody), fixed, permeabilized and stained for intracellular p24 (KC57-RD1 antibody). Columns represent relative MHC-I-mCherry surface expression calculated from the geometric mean fluorescent intensity (gMFI) of surface MHC-I-mCherry on infected cells and normalized to the cell surface MHC-I-mCherry levels of ΔNef-infected cells. Error bars were calculated from four independent repeats. (* indicates *p*-value < 0.01).

The Nef-dependent endocytosis of cell surface MHC-I during an HIV-1 infection leads to evasion of CTL killing, thereby contributing to continued viral replication [38]. Nef-mediated MHC-I downregulation requires subversion of multiple membrane trafficking regulators, including binding to PACS-1 [7, 9, 10, 12, 13, 39]. Therefore, to evaluate the simultaneous expression of Nef with PACS-1 or MHC-I, specifically the HLA-A2 allele, we engineered constructs containing different fluorophores for all genes inserted in the 5’ or 3’ MCS, respectively (Fig 3A). The resulting vectors (pNL4-3 F2A-MHC-I-mCherry Nef-eGFP and pNL4-3 F2A-PACS-1-mCherry Nef-eGFP) were designed to include an mCherry tag at the 5’ site and an eGFP tag at the 3’ site. To confirm the presence of the different fluorophores fused to proteins of interest, we infected HeLa cells with pseudovirions encoding the MHC-I or PACS-1 genes and visualized cells by widefield fluorescence microscopy. This demonstrated simultaneous expression of both mCherry tagged MHC-I or PACS-1 and eGFP tagged Nef (Fig 3B, column 2 and 3). Similar infection of Jurkat E6.1 T-cells with pseudovirions generated from the respective vectors confirmed their simultaneous expression by western blot (Fig 3C, lane 4 and 5). Importantly, the conjugation of mCherry to MHC-I did not alter its localization within the cell as flow cytometry measurements indicated that mCherry-tagged MHC-I expressed in Jurkat E6.1 T-cells infected with the pNL4-3 F2A-MHC-I-mCherry ΔNef pseudovirus, was correctly routed to the cell surface (Fig 3D and S1 Fig). Moreover, MHC-I-mCherry was sensitive to Nef activity as Jurkat E6.1 T-cells infected with pNL4-3 F2A-MHC-I-mCherry Nef-eGFP resulted in less MHC-I-mCherry on the cell surface compared to cells infected with pNL4-3 F2A-MHC-I-mCherry ΔNef (Fig 3D and S1 Fig). These results indicate that co-expression of PACS-1-mCherry or MHC-I-mCherry and Nef-eGFP is achievable. Moreover, using different fluorophores for Nef and its binding partners allows for simultaneous observation of both proteins in infected cells without compromising their functionality.

### Viral BiFC demonstrates that Nef interacts with PACS-1 in specific endosomal compartments

**Fig 4:**
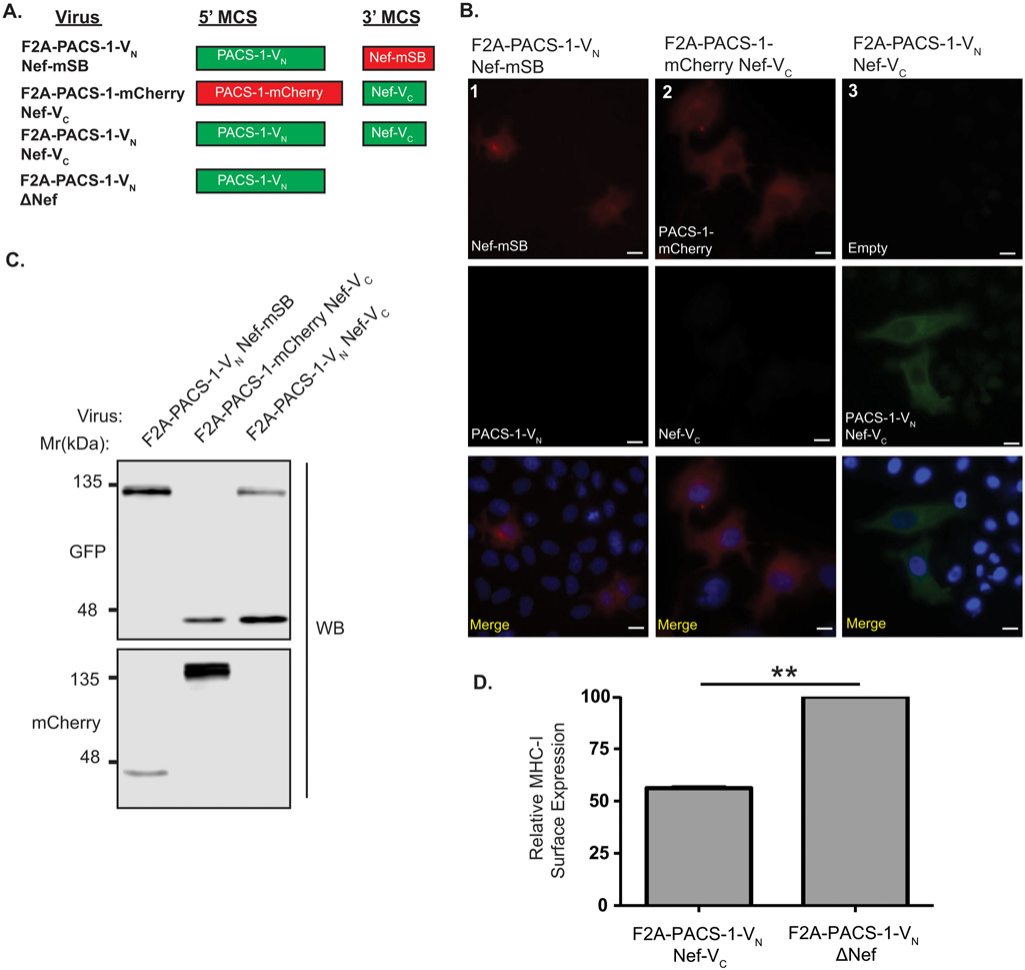
Visualizing the Nef/PACS-1 interaction using viral Bimolecular Fluorescence Complementation. **(A)** Schematic representation of proteins produced from lentiviral expression system. **(B)** HeLa cells were infected with various viruses encoding different fusion proteins and visualized using widefield fluorescence microscopy. BiFC (green, column 3) was visualized in the F2A-PACS-1-V_N_ Nef-V_C_ infected HeLa cells and not the control BiFC viral infections (columns 1 and 2). Cells were mounted in DAPI Fluoromount G media for nuclear staining (blue). Scale bars represent 15μm. **(C)** Expression of the V_N_ or V_C_ fragment was detected by a GFP specific Western blot, whereas the mCherry and mSB fusions, which acted as controls, were detected by an mCherry specific western blot. Densitometry measurements for PACS-1-V_N_ and Nef-V_C_ were 10,500 and 29,200 arbitrary units, respectively, as determined by Licor C-Digit. **(D)** Jurkat E6.1 T-cells were infected with F2A-PACS-1-V_N_ Nef-V_C_ and the corresponding non-functional Nef mutant (F2A-PACS-1-V_N_ ΔNef). At 72 hours post infection, cells were surface stained for MHC-I (W6/32 antibody), fixed, permeabilized and stained for intracellular p24 (KC57-RD1 antibody). Columns represent relative MHC-I surface expression calculated from the geometric mean fluorescent intensity (gMFI) of surface MHC-I on infected cells and normalized to cell surface MHC-I levels of ΔNef-infected cells. Error bars were calculated from four independent repeats. (* indicates *p*-value < 0.01).

To investigate the utility of our lentiviral vector system for studying protein-protein interactions, we constructed lentiviral BiFC vectors (Fig 4A). These were designed such that a functional Venus fluorophore was reconstituted when proteins fused to the amino (V_N_ [1–173]) and carboxy (V_C_ [155–238]) fragments of Venus were in close proximity. Indeed, vectors were designed to contain PACS-1-V_N_ in the 5’ MCS and Nef-V_C_ in the 3’ MCS (pNL4-3 F2A-PACS-1-V_N_ Nef-V_C_). Strikingly, infection of HeLa cells revealed that PACS-1 and Nef reconstitute a functional fluorophore when expressed from the pNL4-3 F2A-PACS-1-V_N_ Nef-V_**C**_ vector, indicating that PACS-1 and Nef are expressed and are in close proximity (Fig 4B, column 3). Analysis at the protein level revealed PACS-1-V_N_ and Nef-V_**C**_ are both expressed (Fig 4C). To rule out possible auto-fluorescence of the individual split fluorophores, we also infected HeLa cells with viruses expressing the individual split fluorophores (Nef-V_C_ or PACS-1-V_N_; Fig 4B), in combination with either PACS-1-mCherry or Nef-mSB (Fig 4A). Indeed, sole expression of PACS-1-V_N_ or Nef-V_C_ did not result in fluorescence (Fig 4B; column 1, 2), even though these proteins were efficiently produced (Fig 4C) and red fluorescence was observed indicating protein expression from the other MCS (Fig 4B; column 1, 2). Moreover, to determine if the Nef protein produced from our viral BiFC vector system was functional, we used flow cytometry to test the ability of Nef produced from pNL4-3 F2A-PACS-1-V_N_ Nef-V_C_ to downregulate endogenous MHC-I in Jurkat E6.1 T-cells. Our analysis demonstrated that Nef-V_C_ downregulated MHC-I efficiently when compared to Jurkat E6.1 T-cells infected with a virus lacking Nef (pNL4-3 F2A-PACS-1-V_N_ ΔNef) (Fig 4D and S2 Fig).

**Fig 5:**
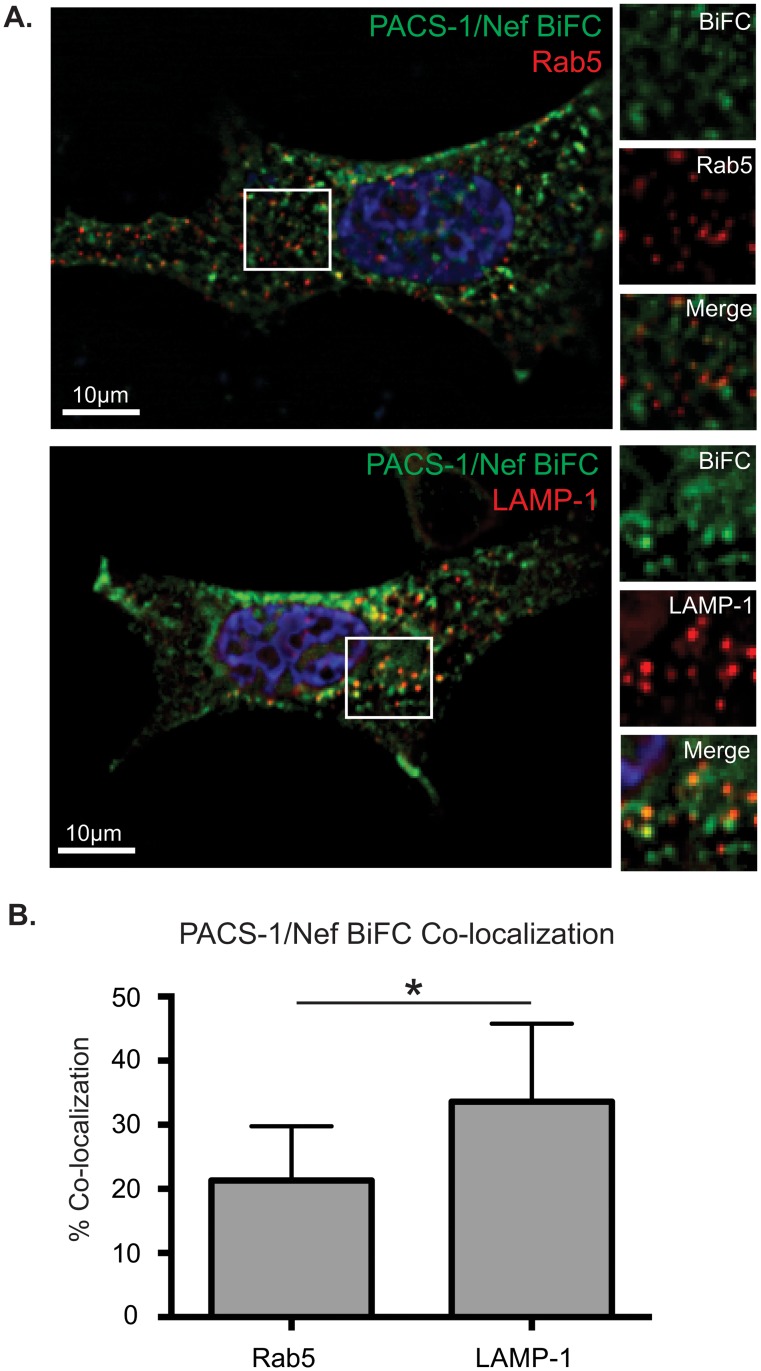
Nef/PACS-1 viral BiFC signal is localized to specific Rab5 and LAMP-1 positive endosomes. **(A)** HeLa cells were infected with the F2A-PACS-1-V_N_ Nef-V_C_ virus and immunostained for Rab5 or LAMP-1. Cells were fixed, permeablized and stained using Rab5 or LAMP-1 specific primary antibodies. Viral BiFC (green) was observed under the FITC channels and Rab5/LAMP-1 (red) fluorescence was observed under the Far-Red channel. Cells were mounted in DAPI Fluoromount G media for nuclear staining (blue). Scale bars represent 10μm. Panels on the right represent a magnification of the boxed region from the left panel. **(B)** Twenty-one percent of the viral BiFC signal co-localized with Rab5, whereas 34% co-localized with LAMP-1. Co-localization was determined by the Manders Coefficient. Pearson’s correlation values were determined to be 0.36 and 0.42 for Rab5 and LAMP-1 co-localization, respectively. Error bars were calculated from 3 independent experiments and quantification of at least 25 different cells. (* indicates p value < 0.05).

To test if the viral BiFC signal between PACS-1-V_N_ and Nef-V_**C**_ was localized to a specific sub-cellular compartment, we performed an immunofluorescence analysis (Fig 5A) using markers of the endocytic pathway previously identified as co-localizing with Nef/PACS-1 complexes [9]. HeLa cells infected with pNL4-3 F2A-PACS-1-V_N_ Nef-V_C_ and exhibiting viral BiFC were stained with markers for late or early endosomes. We observed 21% and 34% co-localization with makers for Rab5 and LAMP-1, consistent with a Nef/PACS-1 interaction on early and late endosomes, respectively (Fig 5B). Together these experiments indicate that viral BiFC can be applied to study protein-protein interactions between Nef and host cellular binding partners, and is particularly effective for mapping the sub-cellular locations of their interactions during viral infection. In addition, viral BiFC produces a functional Nef protein that has the ability to downregulate MHC-I.

### Viral BiFC can be used to study novel interactions between Nef and host cellular proteins in the endocytic network

**Fig 6:**
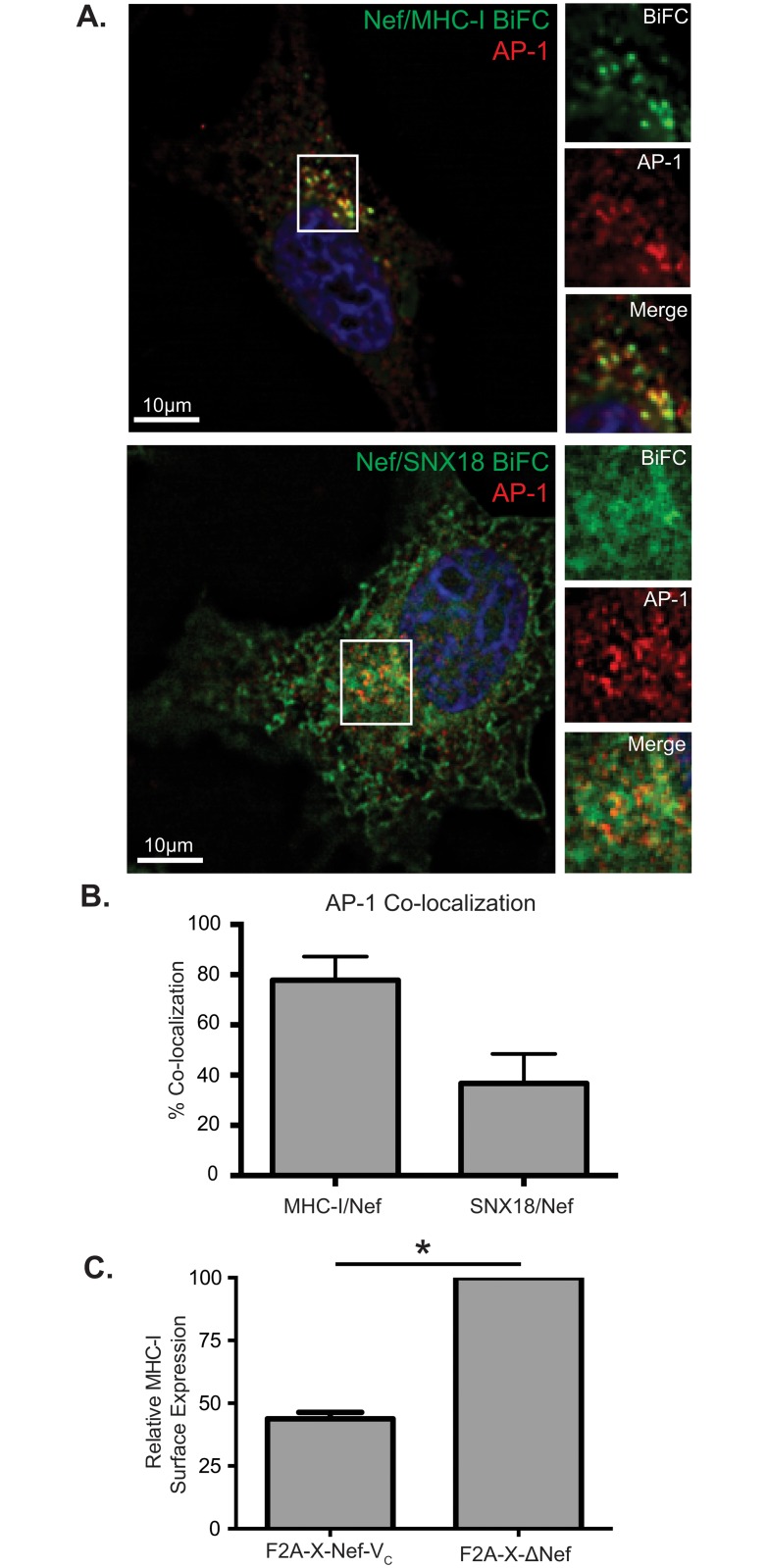
Viral BiFC signals of MHC-I/Nef and SNX18/Nef are localized to AP-1 positive endosomes. (A) HeLa cells were infected with either the F2A-MHC-I-V_N_ Nef-V_C_ virus (top) or the F2A-SNX18-V_N_ Nef-V_C_ virus (bottom) and immunostained for AP-1. Cells were fixed, permeabilized and stained using an AP-1 specific primary antibody. Viral BiFC fluorescence (green) was observed under the FITC channels and AP-1 fluorescence (red) was observed under the Far-Red channel. Cells were mounted in DAPI Fluoromount G media for nuclear staining (blue). Scale bars represent 10μm. Panels on the right represent a magnification of the boxed region from the left panel. (B) 78% percent of the Nef/MHC-I BiFC signal co-localized with AP-1, whereas 37% of the Nef/SNX18 BiFC signal co-localized with AP-1. Co-localization was determined by the Manders Coefficient, and mean Pearson’s correlation was determined to be 0.74 and 0.40 for Nef/MHC-I and Nef/SNX18, respectively. Error bars were calculated by 3 independent experiments and quantification of at least 25 different cells. (C) Jurkat E6.1 T-cells were infected with F2A-X Nef-V_C_ virus and the corresponding non-functional Nef mutant (F2A-X ΔNef). At 72 hours post infection, cells were surface stained for MHC-I (W6/32 antibody), fixed, permeabilized and stained for intracellular p24 (KC57-RD1 antibody). Columns represent relative MHC-I surface expression calculated from the geometric mean fluorescent intensity (gMFI) of surface MHC-I on infected cells and normalized to the cell surface MHC-I levels of ΔNef-infected cells. Error bars were calculated from four independent repeats. (** indicates *p*-value < 0.01).

We demonstrated that viral BiFC can recapitulate previously characterized interactions between Nef and host cellular proteins, such as the Nef/PACS-1 interactions, and can be used to examine the sub-cellular localization of such interactions (Fig 5). Therefore, we decided to test if viral BiFC can spatially define the interaction between Nef and additional interacting partners. We first tested the ability of Nef to interact with the cell surface receptor MHC-I. Indeed, the Nef/MHC-I interaction has been demonstrated both *in vitro* and by crystallography, but this complex has never been demonstrated within cells. Thus, we inserted the MHC-I allele HLA-A2 into the 5’ MCS of our viral BiFC vector to generate the vector pNL4-3 F2A-MHC-I-V_N_ Nef-V_C_. To test for an interaction between MHC-I-V_N_ and Nef-V_C_, HeLa cells were infected and observed under widefield fluorescence microscopy. Interestingly, BiFC was observed, demonstrating an interaction between Nef and MHC-I in cells (Fig 6A). Furthermore, we co-localized this interaction to vesicles that are positive for the adaptor coat protein-1 (AP-1; Fig 6B) consistent with the *in vitro* Nef/MHC-I/AP-1 crystal structure [14].

Since both PACS-1 and AP-1 are implicated in the Nef mediated downregulation of MHC-I, we explored the possibility that Nef associates with another host cellular membrane trafficking regulator, sorting nexin 18 (SNX18). We pursued SNX18 since this protein co-localizes with both PACS-1 and AP-1 within the endocytic network [40]. To test if there is an association between Nef and SNX18, we inserted the *SNX18* gene in the 5’ MCS of our viral BiFC vector to produce pNL4-3 F2A-SNX18-V_N_ Nef-V_C_. We then infected HeLa cells with pNL4-3 F2A-SNX18-V_N_ Nef-V_C_ and tested for BiFC. As for our previously tested interactions, BiFC was observed between SNX18 and Nef demonstrating for the first time the close proximity between Nef and SNX18 (Fig 6A; bottom panels). Furthermore, this association co-localized with AP-1, consistent with SNX18’s localization in the endosomal network (Fig 6) [40]. Importantly, infection with a vector harboring an empty 5’ MCS (pNL4-3 F2A-X Nef-V_C_) demonstrated that Nef-V_C_ expressed from this vector can downregulate endogenous MHC-I in Jurkat E6.1 T-cells (Fig 6C and S3 Fig). This indicates that Nef fused to a split fluorophore is functional. Overall, viral BiFC can be used to further study previously described interactions as well as identify novel interactions between Nef and host cellular partners, and spatially define these interactions within the cell.

## Discussion

This study describes viral BiFC, a novel lentiviral expression system designed for studying protein-protein interactions and mapping their sub-cellular locations. This vector has the unique capability of enabling the use of bimolecular fluorescence complementation from a single vector in the context of a viral infection. We demonstrate the application and utility of viral BiFC for understanding the membrane trafficking pathways of cellular proteins interacting with the HIV-1 protein Nef (Figs 5 and 6). This is of utmost importance as Nef is considered the pathogenic factor responsible for the progression to AIDS [41]. Moreover, since Nef lacks any enzymatic activity its ability to interact with cellular partners is key to define its pathogenic nature.

Lentiviral vectors are ideal vehicles for introducing genes at high expression levels inside a heterologous cell {for review see [18, 42, 43]}. However, the study of protein interactions often requires gene expression from multiple vectors, which may result in suboptimal or differential expression of the proteins of interest. One approach to express multiple genes with lentiviral vectors involves using an internal ribosome entry site (IRES) [44]. However, significant differences in IRES activity have been reported in different cell types, thereby decreasing the utility of these vectors [45, 46]. Moreover, the use of an IRES is limited, as there are significant restrictions imposed when cloning due to the substantial size of IRES sequences [23] and multiple reports suggest certain cistrons inhibit IRES element activity [47, 48].

In contrast, effective protein expression from polycistronic RNA can be achieved using plasmids harboring the 2A peptide sequence, such as in this study, which permits independent translation of coding sequences from a single transcript [23, 49, 50]. This approach allowed for co-expression of PACS-1-mCherry or MHC-I-mCherry and Nef-eGFP, demonstrating that Nef and host cell proteins can be efficiently expressed from a single integrating vector (Fig 3). We validated the cleavage efficiency of the 2A site by constructing a modified vector with a mutated F2A site (Fig 2). Production of cleaved mStrawberry-Flag was significantly reduced (a six-fold decrease; *p*<0.05) in the vector encoding the mutated F2A site (Fig 2B and 2C), directly confirming the efficiency of cleavage mediated by the F2A site.

BiFC is a valuable tool for visualizing protein-protein interactions within a cell {for review see [15, 16]}. This powerful technique can be used to identify the specific sub-cellular locations where protein interactions occur, and to define protein interaction interfaces by elucidating the residues critical for a protein interaction to occur [9, 15, 16, 51]. In the case of Nef, previous reports have utilized BiFC to demonstrate the dimerization of Nef and to study the interaction between Nef and the host cellular proteins PACS-1 and PACS-2 [9, 51–53]. However, these studies utilized a dual vector expression system, which may result in the need to laboriously optimize the expression levels of two different plasmids in order to observe BiFC [51, 53]. This fine-tuning of plasmid levels is effectively removed in our system, which relies on protein expression from a single vector. In addition, previous studies were not conducted under the conditions of viral infection, precluding generalizability of these studies to the nature of HIV-1 infection. Our single vector expression system has the added advantage that all infected cells express both proteins of interest concomitantly within the context of a viral infection (Figs 3 and 4). This is of particular interest when studying interactions between Nef and PACS-1. Indeed, the Nef/PACS-1 interaction modulates the downregulation of cell surface MHC-I via a mechanism that depends on the length of infection [13]. Our viral BiFC system will facilitate full elucidation of the mechanisms governing the influence of the HIV-1 genome on the time-dependent action of the Nef/PACS-1 interaction through deletion of specific HIV-1 proteins within our viral vector. This molecular dissection will be possible using a single vector system that detects Nef interactions (Figs 5A and 6A) and is functionally capable of downregulating MHC-I (Figs 4D and 6C).

Interestingly, our observation of the Nef/PACS-1 interaction at distinct endosomal compartments (Fig 5) is in accordance with the previously defined sub-cellular localization of this interaction [9], thereby validating that viral BiFC demonstrates protein interactions between Nef and host proteins at *bone fide* cellular compartments. Indeed, this system can define the interacting interfaces between Nef and multiple Nef-binding partners as well as map the sub-cellular location of their interactions. Specifically, the interaction between Nef, MHC-I, and the membrane adaptor protein-1 (AP-1) is critical in orchestrating the downregulation of MHC-I to evade immune detection and this interaction has been studied *in vitro* [39, 54, 55]. However, the exact sub-cellular localization of these interactions currently remains unknown. By inserting the MHC-I gene into our viral BiFC system and co-localizing the Nef/MHC-I interaction to AP-1 positive endosomes we effectively recapitulated the Nef/MHC-I/AP-1 interaction in HIV-1 infected cells (Fig 6). This information is critical to understand the exact molecular players that are subverted by Nef in order to traffic MHC-I molecules from the plasma membrane. A novel membrane trafficking regulator that may play a role in this process is SNX18. Our analysis is the first to demonstrate that SNX18 associates with Nef in an AP-1 positive compartment (Fig 6). This is consistent with the reported presence of SNX18 in compartments that are PACS-1 and AP-1 positive [40]. Future studies will be required to correctly decipher the role of SNX18 in Nef mediated MHC-I downregulation and to confirm if the Nef/SNX18 interaction is direct. An attractive hypothesis is a required role for SNX18 in biogenerating specific vesicles required for Nef to correctly remove MHC-I from the cell surface. This implies that Nef may require the fissiogenic ability of proteins such dynamin, which is recruited by SNX18 to correctly shuttle host proteins such as MHC-I to sub-cellular locales [40].

Although constructed as a robust tool to decipher membrane trafficking networks in HIV-1 infected cells, our vector system also has potential as a drug discovery tool. Recently, Poe *et al*. elegantly demonstrated that BiFC can be utilized for high-throughput screening of small molecule inhibitor libraries for molecules that block specific protein-protein interactions [52]. By performing small-molecule screens of compound libraries in cells infected with our viral BiFC system we will be able to identify compounds that disrupt the interactions between Nef and Nef-binding partners such as PACS-1 or the Nef/MHC-I/AP-1 complex. Our system will allow for identification of novel inhibitors of these interactions in a model of infection, thereby affording us key information about the Nef interaction interfaces that mediate immune evasion during HIV-1 infection.

Due to the essential role Nef plays in HIV-1 pathogenesis, our viral BiFC system was designed to study the many interactions Nef must make to modulate infected cells. However, it will be interesting to determine in future studies if removal of Nef from the 3’ MCS will allow viral BiFC to be applicable to any protein-protein interaction analysis. Given that Nef itself has been determined to play a role in viral replication [56], additional studies are required to determine if Nef can be replaced by a heterologous sequence. Previous studies suggest that viruses harboring a Nef gene deletion could still efficiently replicate suggesting that heterologous sequences can be placed in the 3’ MCS [56]. Moreover, the MCSs in our lentiviral system will not only enable insertion of genes of interest, but also sequences capable of silencing host genes, such as short hairpin RNA sequences [57, 58].

In summary, viral BiFC is a powerful tool that enables the study of vesicular trafficking in the context of HIV-1 infection and provides an efficient method to introduce transgenes directly into a lentiviral vector. Viral BiFC will enable researchers to study Nef interactions at specific sub-cellular locales, thereby elucidating key cellular events that mediate HIV-1 pathogenesis.

## Supporting Information

S1 Fig MHC-I-mCherry is correctly trafficked to the cell membrane and sensitive to Nef. Jurkat E6.1 T-cells were infected with a virus expressing MHC-I-mCherry in the presence (F2A-MHC-I-mCherry Nef-eGFP) or absence (F2A-MHC-I-mCherry ΔNef) of eGFP tagged Nef. At 48 hours post-infection cells were washed and stained for surface HLA-A2 using APC/Cy7-conjugated BB7.2 monoclonal antibody. Cells were then washed, fixed and permeabilized to allow for intracellular staining of p24 (using a PE conjugated anti-p24 antibody).
(EPS)

S2 Fig Nef-V_C_ expressed from a viral BiFC vector expressing PACS-1-V_N_ is able to downregulate MHC-I. Jurkat E6.1 T-cells infected with a virus expressing PACS-1-V_N_ in the presence (F2A-PACS-1-V_N_ Nef-V_C_) or absence (F2A-PACS-1-V_N_ ΔNef) of Nef-V_C_. At 72 hours post-infection cells were washed and stained for surface MHC-I (using an APC/Cy7 conjugated pan-selective monoclonal antibody). Cells were then washed, fixed, permeabilized and stained for intracellular p24 (using a PE-conjugated anti-p24 antibody).
(EPS)

S3 Fig Nef-V_C_ expressed from a base viral BiFC vector is able to downregulate MHC-I. Jurkat E6.1 T-cells were infected with a base viral BiFC virus containing Nef-Vc (F2A-X Nef-V_C_) or a virus that does not produce Nef (F2A-X ΔNef). At 72 hours post-infection cells were washed and stained for surface MHC-I (using an APC/Cy7 conjugated pan-selective monoclonal antibody). Cells were then washed, fixed, permeabilized and stained for intracellular p24 (using a PE-conjugated anti-p24 antibody).
(EPS)
